# Systematic review and meta-analysis: What are the implications in the clinical practice?

**DOI:** 10.1590/2176-9451.20.1.017-019.ebo

**Published:** 2015

**Authors:** Claudia Trindade Mattos, Antônio Carlos de Oliveira Ruellas

**Affiliations:** 1 Adjunct professor, Department of Orthodontics, Fluminense Federal University (UFF); 2 Associate professor, Department of Orthodontics, Federal University of Rio de Janeiro (UFRJ)

Systematic reviews and meta-analyses, as already reported in the opening article of this section, are the highest level of scientific evidence^1^ and may strengthen clinically useful evidence.^2^ That means that, according to evidence-based Dentistry, clinically significant results found in clinical and laboratorial researches will be incorporated in clinical practice, especially in dependence on conclusions drawn from systematic reviews and meta-analyses. For this reason, assessing articles and understanding their findings may be a valuable time saver for every clinician who wishes to introduce new conducts, technologies or treatments into his/her clinical practice in a responsible and scientific manner. Therefore, reading a good systematic review or meta-analysis would prevent clinicians from reading several original research articles (which may be gathered in the systematic review) with a view to reaching a clinical conclusion. However, it is critical for the clinician to read systematic reviews and meta-analyses with a minimum comprehension of their structure and characteristics in order to interpret their findings in a reliable and clinically advantageous manner.

Systematic reviews are defined as scientific investigations that attempt to answer focused questions and present pre-planned strategies to include all relevant articles, appraise primary trials and synthesize data, making an effort to reduce bias,^3^ that means, any tendency usually present in research methodology which may lead to misinterpretation of results. As the name suggests, a systematic review should be performed systematically, with transparency in the report of each step in such a way as to minimize subjectiveness.

Meta-analysis may be defined as a statistical synthesis of data^4^ obtained from original research articles previously gathered by means of a systematic review in which data are comparable. Obviously, since meta-analyses are based on statistical data, they have greater scientific evidence power than a systematic review alone. Systematic reviews and/or meta-analyses should be based on a focused question ideally intended to solve a clinical problem. This question may be described in a PICO/PECO format (Patient/Problem/Population; Intervention/Exposure; Comparison and Outcomes). Therefore, the question should be well constructed in clinical terms, approaching the type of population to be studied. In other words, if the authors will approach a specific group of patients in terms of age, sex or race (P); which type of treatment or intervention/exposure in health is to be investigated (I/E); whether this intervention or exposure should be compared to another kind of treatment or to no intervention at all (control groups) (C); and, finally, the ideal outcomes to be obtained by treatment or exposure (O).

Ideally, systematic review authors should report the review goals and research methods used to assess articles by means of registering a protocol in specific databases prior to the beginning of the review. This would avoid potential bias in the review process. Bias could be due to review authors' prior knowledge of the results yielded by potentially eligible studies.^5^ These specific databases are organizations that work for publication and registration of systematic reviews in health, such as PROSPERO (http://www.crd.york.ac.uk/PROSPERO/), associated with the National Institute for Health Research, or the Cochrane Collaboration (http://www.cochrane.org/).

The methods used to carry out a systematic review must be thoroughly described, including how the authors performed search and study selection, the databases searched and the search strategy for each database, particularly reporting the keywords used, the languages in which articles were published and the time period investigated. It is important to emphasize that the methods described should be reproducible. Therefore, inclusion criteria must be clear. After articles have been obtained, they should be selected by means of eligibility criteria which should also be clearly described^3^ in a logical and coherent manner according to the question asked. At this point, the inclusion of a flow diagram is highly recommended in the systematic review description with a view to demonstrating the number of articles obtained and excluded in the selection process. Two reviewers should perform this step independently and their results should be compared, deciding discrepant cases by consensus or with the help of a third reviewer.

Describing the risk of bias or tendency in the included studies is deemed necessary so that the reader may assess the quality of the articles published about a particular theme. To this end, there are several scales, checklists and other tools proposed for assessment. Cochrane Collaboration encourages the use of a specific tool assessing random sequence generation, that is, identifying whether randomization was adequate; allocation concealment; blinding of participants and personnel; blinding of outcome assessment; incomplete outcome data; selective outcome reporting and 'other source of bias'.^5^ The results from the systematic review may be presented in different ways. If a meta-analysis could not be performed due to an insufficient number, in statistical terms, of clinical trials with good scientific evidence, the results and characteristics from each study may have been presented in tables. 

**Figure 1 -:**
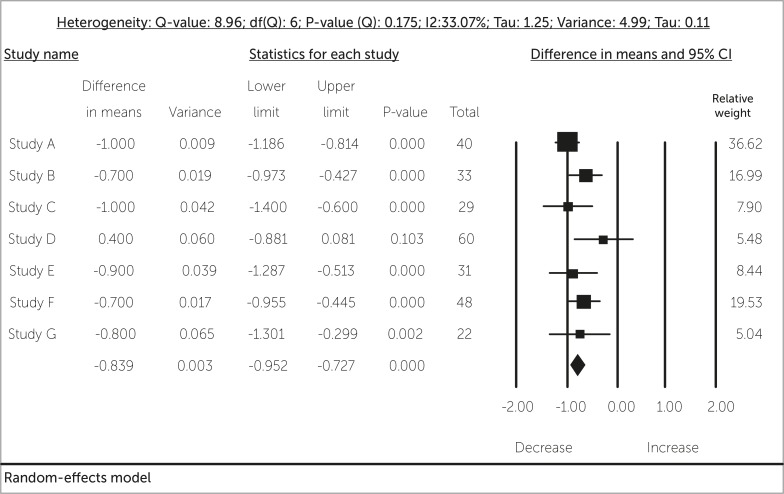
Hypothetical forest plot.

Results from meta-analysis; however, should be presented in figures known as forest plots. A forest plot consists in a graphic that illustrates the statistical weight related to the results of each clinical trial presented in a meta-analysis.

A systematic review author should discuss and interpret the results yielded by the review, its limitations, clinical implications and recommendations. In many systematic reviews, there are no statistically significant conclusions that direct clinical decisions due to the impossibility of gathering data from different studies or due to an insufficient number of studies or patients, which prevents the performance of a meta-analysis. This situation will indicate the need for better clinical trials, in scientific terms, in order to answer the clinical question. On the contrary, when the specific initial clinical question has been answered, either positively or negatively considering a determined treatment or exposure, that means there is no more scientific need for further clinical trials on that specific point, preserving ethically future research subjects and directing research funding to other clinical questions in health.

Finally, according to Feldstein,^3^ systematic reviews will continue to play a major role in translating research evidence into patient care decisions, directing our clinical practice based in scientific evidence and indicating new research themes, thereby contributing to science growth in response to health care. 
